# Heart Failure With Type 2 Diabetes Mellitus: Association Between Antihyperglycemic Agents, Glycemic Control, and Ejection Fraction

**DOI:** 10.3389/fendo.2020.00448

**Published:** 2020-07-10

**Authors:** Shu Ning Lin, Kok Kit Phang, Seng Hsiung Toh, Kok Han Chee, Hasniza Zaman Huri

**Affiliations:** ^1^Department of Medicine, Faculty of Medicine, University of Malaya, Kuala Lumpur, Malaysia; ^2^Department of Medicine, Hospital Tengku Ampuan Rahimah, Ministry of Health Malaysia, Klang, Malaysia; ^3^Department of Cardiology, Hospital Queen Elizabeth II, Ministry of Health, Kota Kinabalu, Malaysia; ^4^Department of Clinical Pharmacy and Pharmacy Practice, Faculty of Pharmacy, University of Malaya, Kuala Lumpur, Malaysia

**Keywords:** antihyperglycemic agent, glycemic control, ejection fraction, heart failure, diabetes

## Abstract

**Background:** Heart failure (HF) is associated with type 2 diabetes mellitus (T2DM). Antihyperglycemic drugs have interaction with heart failure among diabetic patients. To date, the data on real world use of diabetic medication in Malaysian heart failure patients with T2DM has not been elucidated.

**Objective:** This study aims to identify the prescribing pattern of antihyperglycemic regimens in HF patients with T2DM, and to investigate the association between glycemic control and other factors such as demographic and clinical characteristics with left ventricular ejection fraction (LVEF) in these patients.

**Methods:** This retrospective observational study involved patients diagnosed to have HF and T2DM who were seen in the outpatient clinic in a government tertiary hospital in Malaysia. Patients receiving at least one oral antidiabetic agent and/or insulin for at least 3 months prior were included. The differences and association between study outcomes were examined and analyzed using Pearson's Chi-square test, One-Way ANOVA, Binary Logistic Regression and multiple Multinomial Logistic Regression models.

**Results:** From July to December 2019, 194 patients were included in this study. The majority (52.1%) of the patients had HF with preserved ejection fraction (HFpEF), 20.6% had HF with mid-range EF (HFmrEF), and 27.3% had HF with reduced EF (HFrEF). Overall, metformin (59.8%) was the commonest antihyperglycemic agent prescribed, followed by insulins (54.0%), and sulphonylureas (44.9%). The most prescribed agents for HFpEF, HFmrEF, and HFrEF patients were metformin (65.3%), insulins (62.5%), and sulphonylureas (60.4%), respectively. The prescribing trend of sulphonylureas was found to be significantly associated with patients' LVEF status (*p* = 0.033). The odds for sulphonylurea prescription among the HFrEF patients were 2.42 times higher compared to the HFpEF patients [95% confidence interval [CI], 1.23–4.79]. There was no association found between glycemic control with patients' LVEF.

**Conclusion:** Our findings reported metformin as the most commonly prescribed antihyperglycemic agent, sodium glucose linked transporter-2 (SGLT-2) inhibitor being under-prescribed, and detected poorly controlled diabetes in majority of patients with T2DM and HF. Understanding the prescribing pattern of antihyperglycemic agents supports the implementation of evidence-based treatment in HF patients with T2DM to improve patients' outcomes.

## Introduction

Heart Failure (HF) is a progressive and chronic clinical syndrome characterized by typical symptoms of breathlessness, orthopnea, or ankle swelling, caused by a structural or functional cardiac abnormality, resulting in a decreased cardiac output and raised intracardiac pressures at rest or during stress (1). Heart failure is a global pandemic affecting at least 26 million people worldwide and is increasing in prevalence (2). The prevalence of heart failure ranges between 3 and 20 per 1,000 population (3).

Type 2 diabetes mellitus (T2DM) is a prevalent non-communicable disease characterized by hyperglycemia resulting from the combination of defects in insulin secretion, resistance to insulin action, and excessive glucagon secretion. The prevalence of T2DM has increased by 30% globally in the past decade (2). In Malaysia, the National Health and Morbidity Survey (NHMS) 2015 has reported an overall diabetes prevalence of 17.5% for adults above the age of 18 years, detecting an increment in the prevalence compared with 15.2% in the year 2011 (4, 5).

T2DM is a well-established risk factor for cardiovascular disease, with heart failure being tightly-linked to T2DM (6). Data from The Framingham Heart Study highlighted that the risks of heart failure are 2- and 5-fold, respectively, in men and women with diabetes (7). Data from the OPTIMIZE-HF and EVEREST studies also reported that ~40% of patients hospitalized with heart failure had a diagnosis of diabetes (8, 9).

Classification of HF patients according to left ventricular ejection fraction (LVEF) has been shown to have prognostic significance. Heart failure has been categorized into three subtypes: HF with preserved Ejection Fraction (HFpEF) patients with LVEF ≥50%, HF with mid-range Ejection Fraction (HFmrEF) patients with LVEF 40–49%, and HF with reduced Ejection Fraction (HFrEF) patients with LVEF <40% (1). LVEF is an established efficacy measure to predict major adverse cardiac events (MACE) (10). A 3% improvement in LVEF was shown to correlate with a 20% improvement in mortality (11). Patients in different classes of LV myocardial dysfunction were also reported to respond to therapies differently. While the 1-year mortality rate after risk-adjustments appeared comparable among patients in different LVEF categories, heart failure patients with increasing New York Heart Association (NYHA) functional class face increased morbidity risk. NYHA functional classification is used to grade and describe the severity of symptoms and exercise tolerance of heart failure patients (1). Higher NYHA classes are associated with increased 1-year mortality rate. The 1-year mortality is estimated to be 5–10, 10–15, 15–20, and 20–50%, respectively, for patients with heart failure NYHA I, II, III, and IV (3).

Poor glycemic control and insulin resistance are associated with deterioration of heart failure and LV dysfunction (2). However, available data suggest no difference in the risk of worsening heart failure between subjecting patients to intensive glycemic control and standard treatment arms (2). The relationship between glycated hemoglobin (Hba1c) and LVEF remains unclear. The U-shaped relationship between HbA1c and mortality among HF patients does not translate into precise glycemic targets or ranges recommended for this group of patient (2).

When choosing appropriate antihyperglycemic therapies in patients with essential comorbidities such as heart failure, a patient-centered approach should be emphasized. Metformin have been shown to be safe and effective. On the contrary, insulin and sulphonylurea have been associated with worsening of heart failure (1). Thiazolidinediones have also demonstrated a consistent and robust relationship with increased risk of heart failure (12). Pooled analysis of currently available Cardiovascular Outcome Trials (CVOTs) of glucose-lowering medications reported that dipeptidyl peptidase-4 inhibitor (DPP-4i) and GLP1-RA resulted in a neutral effect on heart failure hospitalization in patients with T2DM. In contrast, sodium glucose linked transporter-2 (SGLT-2) inhibitor resulted in a statistically significant reduction in heart failure hospitalization (13). While suggesting that metformin and sulphonylurea being generally safe, the Malaysian Clinical Practice Guideline (CPG) on the Management of Heart Failure (HF) also recommends SGLT-2 inhibitor in the treatment of diabetes in patients with HF (3).

The current published CVOTs of antihyperglycemic agents involve a large number of subjects from Asians, Caucasians, and Blacks patients, across different countries. Translating clinical trial data to real world practice, especially in resource-limited countries or hospitals, remains a challenge. The data on real world use of diabetic medicine in Asian HF patients with T2DM are not well-documented. This underscores the importance of seeking a better understanding of ethnicity-tailored treatment strategies for heart failure patients with T2DM.

Thus, a localized clinical observational study is required to identify the prescribing pattern of different classes of antihyperglycemic therapies, as well as to examine the effect of patients' glycemic control status on patients' cardiac function, in a real-world population of patients with both the diseases. This study aims to assess the prescribing pattern of antihyperglycemic regimens in heart failure patients with type 2 diabetes mellitus, and to investigate the association between glycemic control and other factors such as demographic and clinical characteristics, with LVEF of these patients.

## Methodology

### Study Design and Setting

This retrospective observational study was conducted at Hospital Tengku Ampuan Rahimah (HTAR) Klang, a government tertiary hospital with 831 beds. HTAR caters to a multi-ethnic society that makes up the population of Malaysia. The study was conducted in compliance with the ethical principles outlined in the Declaration of Helsinki and Malaysian Good Clinical Practical Guideline. Ethical approval has been obtained from the Medical Research and Ethics Committee (MREC), Ministry of Health Malaysia [Reference number NMRR-19-1358-48105 (IIIR)]. The MREC of MOH waived the need for written informed consent from the participants.

### Inclusion and Exclusion Criteria

All eligible patients who fulfill the following inclusion and exclusion criteria were included in this study. The inclusion criteria were patients aged ≥ 18 years old, diagnosed to have heart failure based on clinical diagnosis with echocardiographic evidence of ejection fraction ≤ 70%, diagnosed with type 2 diabetes mellitus with laboratory measured HbA1c results, and prescribed with at least one oral antidiabetic agent and/or insulin and receiving antidiabetic therapy for at least 3 months prior to enrolment. The exclusion criteria were patients without an echocardiography report and HbA1c results, T2DM patients who were not receiving any antidiabetic agent and managed with diet controls and lifestyle modification, and pregnant women.

### Study Procedure

The potential patients who were coming for follow-up in the Medical Outpatients Clinic, HTAR from July to December 2019 were identified from MOPD Registry. During the patients' visit, an initial screening through patients' medical records for diagnosis of heart failure was conducted. Patients' echocardiography reports were retrieved from Echocardiogram Registry, with echocardiogram tests performed as early as 1 week prior to patients' scheduled clinic appointments. Patients diagnosed with both HF with left ventricular ejection fraction LVEF of ≤ 70% and T2DM were selected.

Data that were collected from patients' medical records included demographic information such as age, sex, ethnicity, weight, height, clinical characteristics including medical history, comorbidities, duration of diabetes, duration of heart failure, LVEF, NYHA classification, relevant laboratory results such as Hba1c, and lastly, medication history, current prescribed diabetes medication regimen, and other concurrent medications.

**Figure 1:**
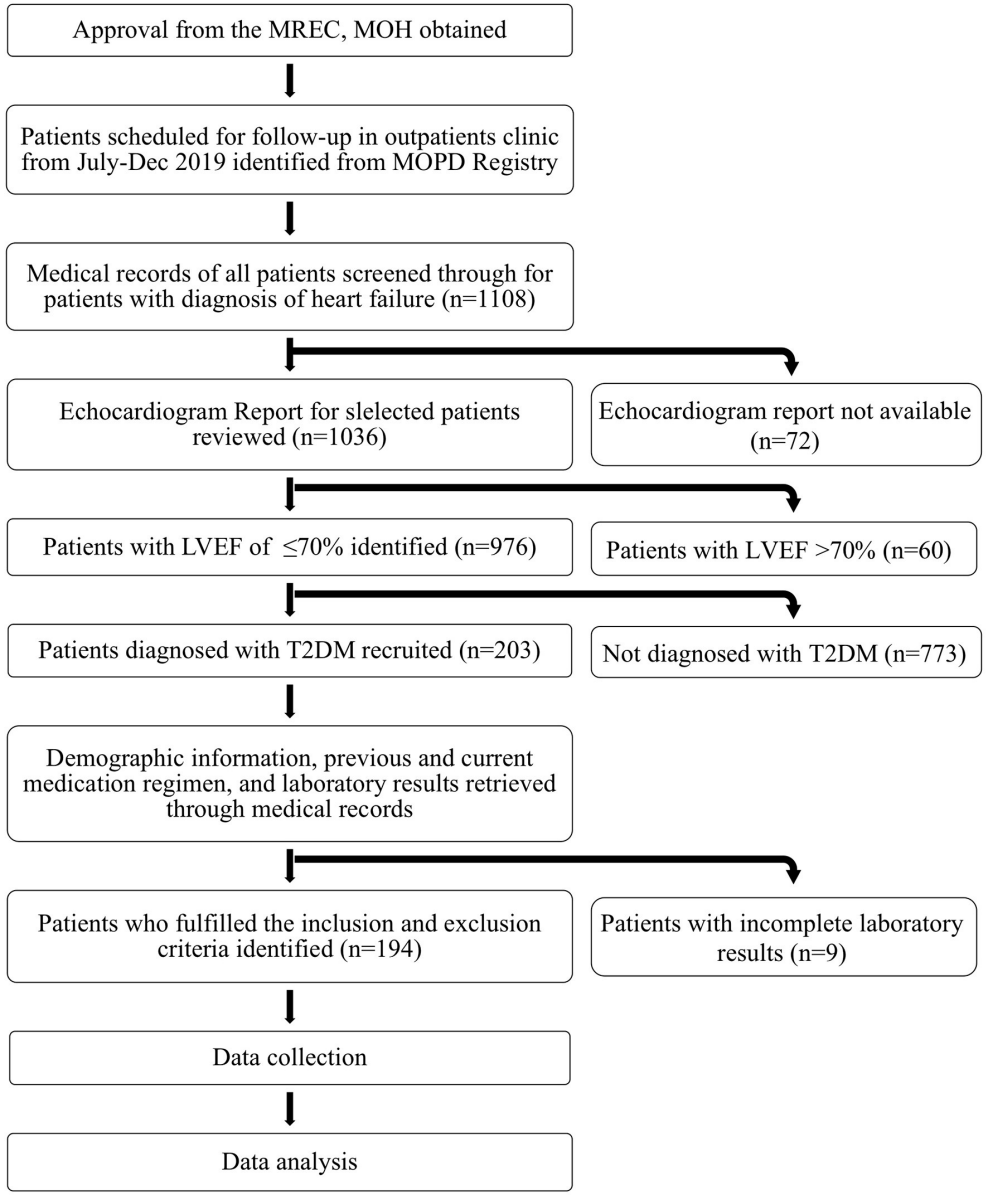
Overview of study design and study subjects' selection.

Finally, patients who fulfilled the inclusion and exclusion criteria were identified and were included in the study, with the summarized findings presented in Figure 1. Patients were then classified according to their LVEF into HF with preserved ejection fraction (HFpEF), HF with mid-range LVEF (HFmrEF), and HF with reduced Ejection Fraction (HFrEF).

Relevant data extracted from medical records were manually entered into the data collection forms. This was done with the permission from the treating physicians, with patients' identities remaining anonymous.

### Study Outcomes

The primary outcome was to identify the prescribing pattern of antihyperglycemic regimens across the three patient groups, which are HFpEF patients, HFmrEF patients, and HFrEF patients. The secondary outcomes were to determine if there was an association between glycemic control and other patient characteristics with LVEF in HF patients with T2DM.

### Sampling Size

The sample size required for this study was estimated using OpenEpi, Version 3.01 (14). Assuming 95% confidence interval or 5% level of significance and 5% margin of error, the minimum sample size calculated was 139.

### Statistical Techniques

Data obtained were analyzed using Statistical Package for Social Science (SPSS) software Version 23 (IBM Corp., Armonk, N.Y., USA). Normality test was done using the Kolmogorov-Smirnov test. Continuous data were presented in mean and standard deviation (SD) if normally distributed, and in median with 25–75% percentile values if non-normally distributed. Categorical data were reported in frequency and percentage. Pearson's Chi-square test was used to study the association between categorical variables. The One-Way ANOVA procedure was used to test for difference in characteristics in the three groups of patients. Odds ratio [95% confidence intervals [CI]] was computed using Binary Logistic Regression with dummy variable. Multiple Multinomial Logistic Regression models were conducted to test the association between antihyperglycemic agent and patients' characteristics with LVEF, adjusting for age, sex, and HbA1c. Significance level was set at *p* < 0.05.

## Results

### Selection of Study Subjects

As illustrated in Figure 1, there were a total of 1,108 heart failure patients who came for follow-up from July-Dec 2019. Out of the 976 HF patients who had echocardiographic evidence of ejection fraction ≤ 70, 20.8% had a concomitant diagnosis of T2DM. Nine patients were excluded from the study for not fulfilling the inclusion criteria. Therefore, a final total number of 194 patients were included in the study.

### Demographic and Clinical Characteristics

Majority (52.1%) of the patients had HFpEF, 20.6% had HFmrEF, and 27.3% had HFrEF. The study population consisted of nearly three-quarters of male. As shown in Table 1, patients' age was normally distributed with a mean ± standard deviation (SD) of 59.6 ± 12.0 years, with a minimum age of 25 and maximum age of 88 years old. LVEF was found to increase with advancing age. Patients from HFrEF group had the youngest mean age (57.8 ± 10.9 years). The largest ethnic population in this study was Malay, followed by Indian, Chinese, and others. When we compared between the three LVEF subgroups, there was significant sex disparity in the population of HF patients with T2DM. More than 80% of male patients were in the HFmrEF and HFrEF categories; while there were more than twice as many females in the HFpEF groups in comparison with HFmrEF or HFrEF groups. Sex was the only characteristic found to be significantly associated with LVEF status. Male HF patients were significantly more likely to have LVEF of ≤ 50% (*p* = 0.002). There were no significant association between other demographic characteristics such as age and ethnicity with LVEF status across the three patient groups (Table 1).

**Table 1:** Demographic and clinical characteristics of patients.

**Demographic and**	**Total**	**HFpEF**	**HFmrEF**	**HFrEF**	***p*-value**
**clinical**	***n* = 194**	***n =* 101**	***n =* 40**	***n =* 53**	
**characteristics**		**(52.1%)**	**(20.6%)**	**(27.3%)**	
Age	59.6 (12.1)	60.5 (12.8)	59.9 (11.5)	57.8 (10.9)	0.429^a^
Sex					
Male	140 (72.2)	62 (61.4)	35 (87.5)	43 (81.1)	**0.002^b^**
Female	54 (27.8)	39 (38.6)	5 (12.5)	10 (18.9)	
Ethnicity					
Malay	78 (40.2)	39 (38.6)	17 (42.5)	22 (41.5)	0.902^b^
Chinese	43 (22.2)	25 (24.8)	7 (17.5)	11 (20.8)	
Indian	70 (36.1)	35 (34.7)	15 (37.5)	20 (37.7)	
Others	3 (1.5)	2 (2.0)	1 (2.5)	0 (0.0)	
LVEF (%)	51.0 (40–65)	64 (56–67)	45 (43–47)	35 (31.5–37)	**<0.001^c^**
NYHA Functional Class					
Class I	153 (78.9)	80 (79.2)	32 (80.0)	41 (77.4)	0.097^b^
Class II	35 (18.0)	20 (19.9)	8 (20.0)	7 (13.2)	
Class III	5 (2.6)	1 (1.0)	0 (0.0)	4 (7.5)	
Class IV	1 (0.5)	0 (0.0)	0 (0.0)	1 (1.9)	
HbA1c (%)	8.2 (6.9–9.5)	8.2 (7.1–9.6)	8.5 (6.8–9.8)	7.8 (6.7–9.1)	0.348^c^
Glycemic Control					
HbA1c ≤ 7	50 (25.8)	22 (21.8)	10 (25.0)	18 (34.0)	0.258^b^
HbA1c >7	144 (74.2)	79 (78.2)	30 (75.0)	35 (66.0)	

*Values are expressed as number (%) for categorical data; mean ± standard deviation, or median (interquartile range) for continuous data*;

a *computed by One-Way ANOVA*;

b *computed by Pearson's Chi-square test*;

c *computed by Kruskal Wallis test; bolded font indicates statistical significance at p < 0.05. HbA1C, glycated hemoglobin; HFmrEF, heart failure with mid-range ejection fraction; HFpEF, heart failure with preserved ejection fraction; HFrEF, heart failure with reduced ejection fraction; LVEF, left ventricular ejection fraction; SGLT-2, sodium glucose linked transporter-2; NYHA, New York Heart Association*.

LVEF was not normally distributed with a median of 51% (interquartile range 40–65%). In the study population, more than half of the patients had LVEF of ≥ 50%. Results revealed that more than three-quarters of patients were placed in NYHA Class I, followed by Class II, III, and IV. In all groups, more than three-quarters of patients were placed in NYHA Class 1. In this study, only one patient, under the HFrEF group, was assigned with NYHA Class IV. Median HbA1c was 8.2% (interquartile range 6.9–9.5%). Approximately three-quarters (74.2%) of patients had poor glycemic control with HbA1c of >7, categorized based on the American Diabetes Association recommendations. Among the three patient groups, a higher proportion of HFrEF patients had good glycemic control and had the lowest median HbA1c. There were no significant differences in terms of NYHA functional class and glycemic control across the three patient groups.

### Antihyperglycemic Agents Prescribed in HF Patients With T2DM

Overall, metformin (59.8%) was the most commonly prescribed antihyperglycemic agent, followed by insulins (56.2%), and sulphonylureas (45.4%), as shown in Table 2. In HFpEF patients, metformin (65.3%) was most commonly prescribed, followed by insulins (60.4%). In HFmrEF patients, insulins (62.5%) were most commonly prescribed, followed by metformin (57.5%). The most commonly prescribed agent for patients with HFrEF was sulphonylurea (60.4%). More than 60% of patients from HFpEF and HFmrEF categories were prescribed with insulins.

**Table 2:** Antihyperglycemic agents prescribed in HF patients with T2DM.

**Classes of**	**Number of patients (%)**				***p*-value^b^**
**antihyperglycemic**					
**agents**					
	**Total^a^**	**HFpEF**	**HFmrEF**	**HFrEF**	
		**(*n =* 101)**	**(*n =* 40)**	**(*n =* 53)**	
Biguanides					
Yes	116 (59.8)	66 (65.3)	23 (57.5)	27 (50.9)	0.211
OR (95% CI)		Reference	0.72 (0.34–1.52)	0.55 (0.28–1.08)	
Sulphonylureas					
Yes	88 (45.4)	39 (38.6)	17 (42.5)	32 (60.4)	**0.033**
OR (95% CI)		Reference	1.18 (0.56–2.47)	**2.42 (1.23–4.79)**	
DPP-4 Inhibitors					
Yes	50 (25.8)	30 (29.7)	9 (22.5)	11 (20.8)	0.420
OR (95% CI)		Reference	0.69 (0.29–1.62)	0.62 (0.28–1.37)	
SGLT2 Inhibitors					
Yes	4 (2.1)	1 (1.0)	1 (2.5)	2 (3.8)	0.501
OR (95% CI)		Reference	2.56 (0.16–42.01)	0.27 (0.35–44.28)	
α-glucosidase Inhibitors					
Yes	1 (0.5)	0 (0.0)	0 (0.0)	1 (1.9)	0.263
OR (95% CI)		UTC	UTC	UTC	
Insulins					
Yes	109 (56.2)	61 (60.4)	25 (62.5)	23 (43.4)	0.086
OR (95% CI)		Reference	1.09 (0.51–2.32)	0.50 (0.26–0.99)	

a *a patient may be prescribed with more than one antidiabetic agent*;

b *computed by Pearson's Chi-square test; odds ratio (95% CI) computed by Binary Logistic Regression with dummy variable; bolded font indicates statistical significance at p < 0.05. α-glucosidase, alpha-glucosidase; CI, confidence interval; DPP-4, dipeptidyl peptidase-4; HFmrEF, heart failure with mid-range ejection fraction; HFpEF, heart failure with preserved ejection fraction; HFrEF, heart failure with reduced ejection fraction; OR, odds ratio; SGLT-2, sodium glucose linked transporter-2, UTC, unable to compute*.

Sulphonylurea was the only antihyperglycemic agent class found to be significantly associated with LVEF subgroups (*p* = 0.033). From Table 2, the odds for sulphonylurea prescription among the HFrEF patients were 2.42 times higher compared to the HFpEF patients, with a 95% confidence interval of [1.23–4.79]. The HFrEF patients were less than half as likely to being prescribed with insulins compared to the HFpEF patients, with a 95% confidence interval of [0.26–0.99]. Otherwise, there were no differences in terms of prescribing pattern of other agents across the three patient groups.

#### Biguanides

**Figure 2:**
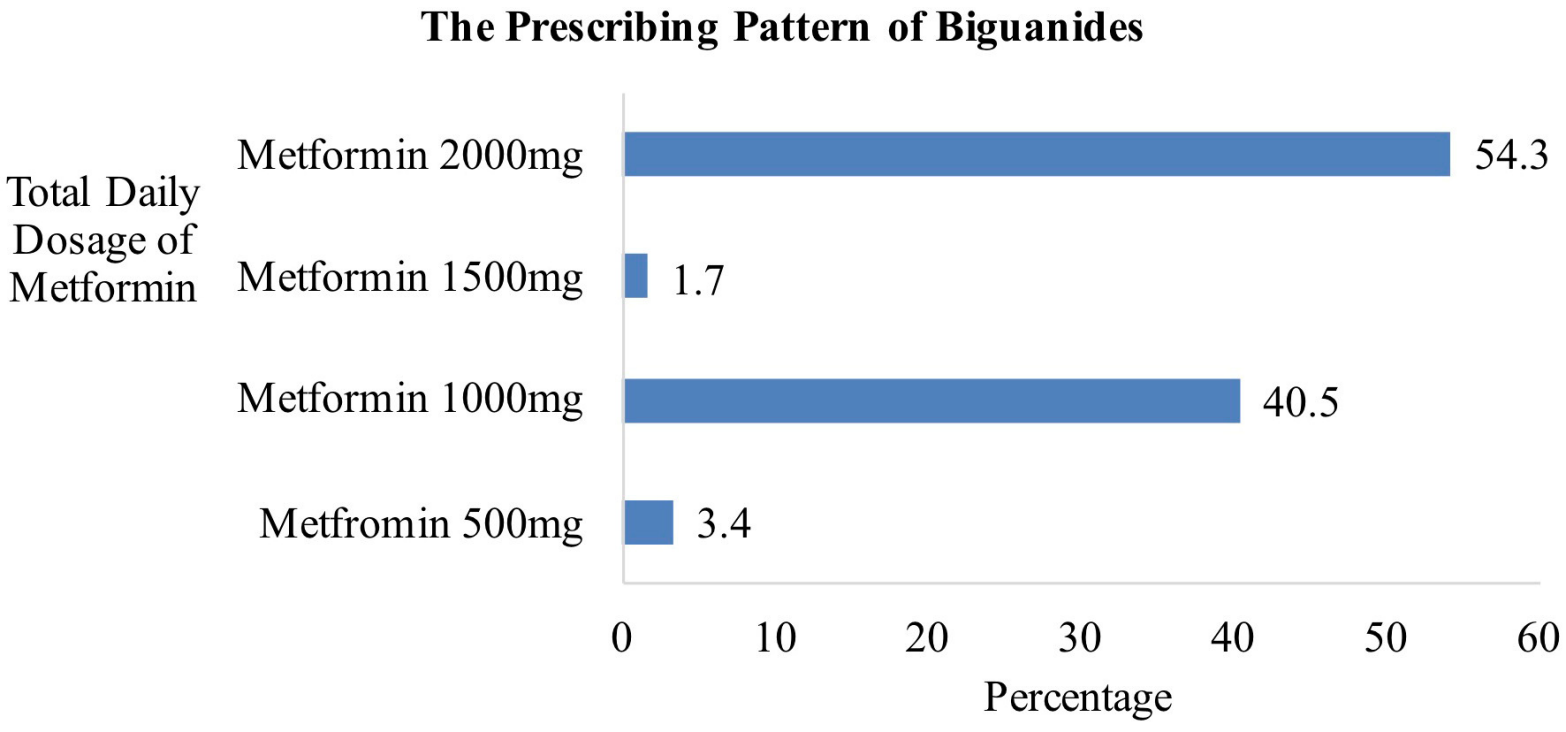
Daily dosage regimens of metformin (*n* = 116).

Metformin was prescribed in 116 patients for glycemic control. As shown in Figure 2, the most frequently prescribed regimen was metformin 2,000 mg daily, in 63 patients. Sixty-one patients were prescribed with immediate-release metformin 1 g twice daily, while two were prescribed with extended-release (ER) metformin at a dose of 2,000 mg once daily. Two patients on metformin 1,500 mg daily were both prescribed with once-daily extended-release tablets. Out of 47 patients receiving metformin 1,000 mg daily, five received metformin ER 1 g once daily while the remaining was on immediate-release metformin 500 mg twice daily. A total of four patients received metformin 500 mg daily, with two patients receiving metformin 250 mg twice daily, and another two receiving metformin ER 500 mg daily.

#### Sulphonylureas

A total of 88 patients were prescribed with gliclazide. Thirty-six patients (40.9%) from them were prescribed with gliclazide modified release (MR) formulation.

#### Dipeptidyl Peptidase-4 Inhibitors

More than 80% of patients on DPP-4 inhibitors were on vildagliptin, with 12 patients on vildagliptin 50 mg od, and 30 patients on vildagliptin 50 mg bd; whereas the remaining eight patients were prescribed with sitagliptin in different doses of 25, 50, or 100 mg once daily.

#### Sodium Glucose Linked Transporter-2 Inhibitors

Only four patients were prescribed with SGLT-2 inhibitors. The dosage regimens used in these patients were once-daily empagliflozin 25, 12.5, and 10 mg.

#### Insulins

**Figure 3:**
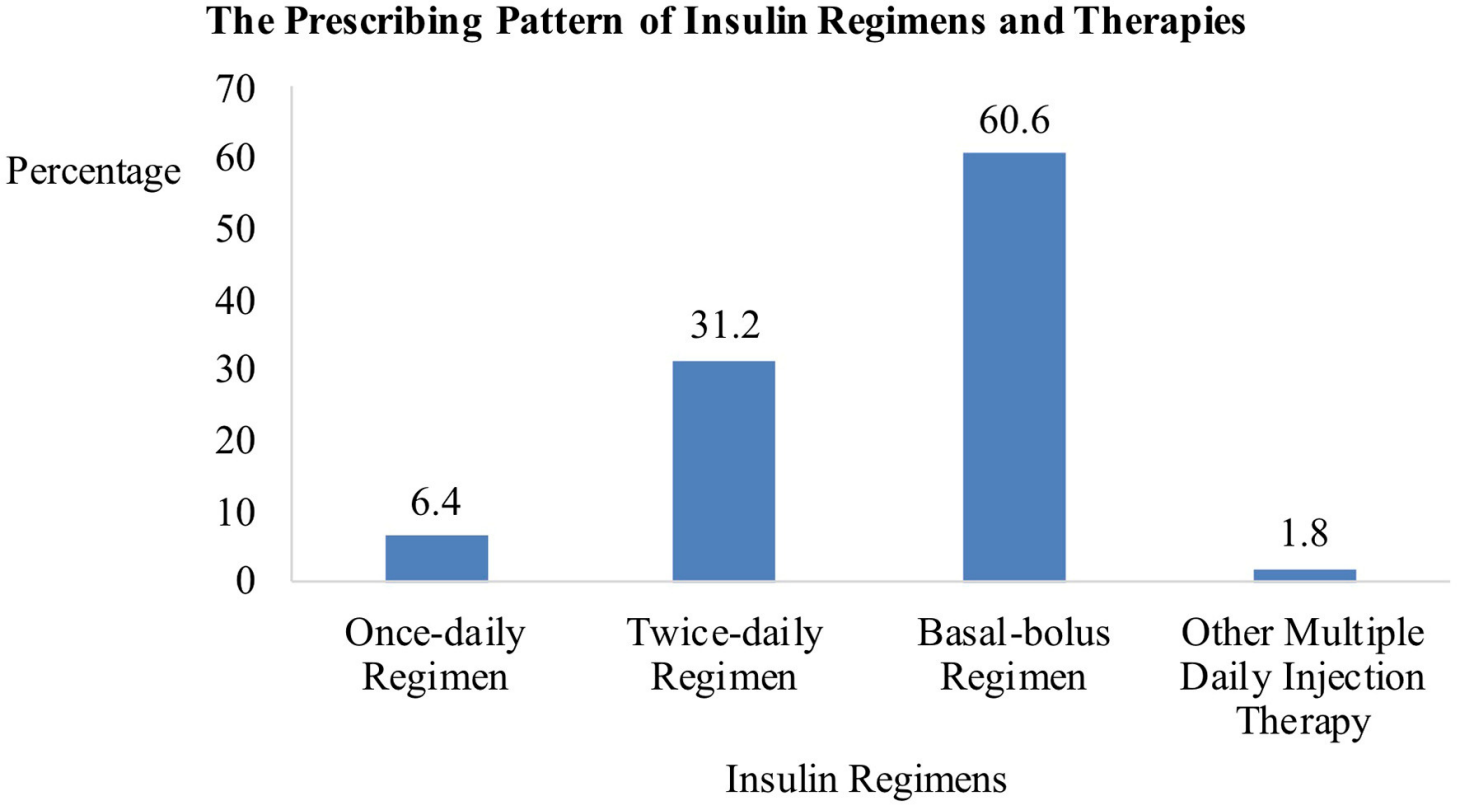
Daily Regimens of Insulins (*n* = 109).

As shown in Figure 3, the subcutaneous basal-bolus regimen was the most commonly prescribed. Sixty-one out of 66 patients on a basal-bolus regimen were prescribed with a combination of intermediate-acting NPH insulin and three separate injections of short-acting regular insulin at each meal. The remaining five patients were prescribed with long-acting basal insulin analogs. The second most frequently prescribed regimen was the twice-daily pre-mixed insulin. All of the 34 patients were prescribed with pre-mixed human insulin except for one who was given pre-mixed insulin analog. Of the seven patients receiving once-daily insulin before bed, six received NPH insulin while one received insulin analog. Two other patients received multiple insulin injection regimens. Overall, more than 90% of patients requiring insulin were prescribed with human insulins instead of insulin analogs.

### Associations of Patients Characteristics and Antihyperglycemic Agents With LVEF

In Table 3, multiple Multinomial Logistic Regression models on the association between sulphonylurea prescribing trend and patients' characteristics with LVEF were displayed. In Model 1, being male was significantly associated to be in the HFmrEF and HFrEF subgroups with ORs of 4.40 (95% CI 1.59–12.20) and 2.71 (95% CI 1.22–6.00), respectively. In Model 2, unadjusted association between sulphonylurea and LVEF subgroups showed that those who were prescribed with sulphonylurea were 2.42 times more likely to be HFrEF patients. Both sulphonylurea prescription and male sex remained significantly associated with HFrEF subgroup even after adjusting for age and HbA1c in the subsequent models.

**Table 3:** Patients characteristics and antihyperglycemic agents associated with LVEF.

	**Odds Ratio (95% CI)**		
	**HFpEF**	**HFmrEF**	**HFrEF**
Model 1
Male Sex	Reference	**4.40 (1.59–12.20)**	**2.71 (1.22–6.00)**
Model 2
Sulphonylurea	Reference	1.18(0.56–2.47)	**2.42 (1.23–4.79)**
Model 3
Male Sex	Reference	**4.40 (1.58–12.25)**	**2.51 (1.11–5.66)**
Sulphonylurea	Reference	1.04 (0.48–2.23)	**2.22 (1.11–4.45)**
Age	Reference	0.99 (0.96–1.03)	0.98 (0.95–1.01)
Model 4
Male Sex	Reference	**4.78 (1.69–13.50)**	**2.35 (1.03–5.39)**
Sulphonylurea	Reference	1.07 (0.49–2.30)	**2.17 (1.08–4.36)**
Age	Reference	0.99(0.97–1.03)	0.98 (0.95–1.01)
HbA1c	Reference	1.11 (0.93–1.31)	0.94 (0.79–1.11)

*Computed by Multinomial Logistic Regression; bolded font indicates statistical significance at p < 0.05. CI, confidence interval; HbA1C, glycated hemoglobin; HFmrEF, heart failure with mid-range ejection fraction; HFpEF, heart failure with preserved ejection fraction; HFrEF, heart failure with reduced ejection fraction*.

## Discussion

In our study cohort of patients with both HF and T2DM, metformin is the most prescribed antihyperglycemic agent. The prescribing trend of sulphonylureas was significantly associated with patients' LVEF status, where HFrEF patients were 2 times more likely to receive sulphonylurea compared to the HFpEF patients. The use of SGLT-2 inhibitors, the evidence-based medications associated with improved outcomes in HF with T2DM, was low at 2.1%. There was no association found between glycemic control with patients' LVEF.

Metformin was the most utilized (59.8%) antihyperglycemic agent as metformin was the recommended first-line medication in the current practice guidelines (15). Metformin was also the most commonly prescribed diabetes medication in the Asian Sudden Cardiac Death in Heart Failure (ASIAN-HF) registry, the Dapagliflozin And Prevention of Adverse-outcomes in Heart Failure (DAPA-HF) trial, and other studies across Asia, with prescription rates ranging from 50.8 to 56.8% (16–18). The prescription rates reported were similar to that in our study. In contrast, two individual Asian countries namely Japan and China reported low metformin use at 11.8 and 14.5%, respectively, for reasons yet to be assessed (17). The European Society of Cardiology (ESC) Guidelines for heart failure reported prescribing of metformin to be safe for HF patients, taking into consideration the patient's age and degree of renal or hepatic dysfunction (1). Metformin is effective, safe, and associated with improved outcomes such as reduction in all-cause hospitalization and mortality in HF patients in the ASIAN-HF registry (19, 20). Therefore, in accordance with current guideline recommendations, metformin should remain as the first drug of choice unless contraindicated.

Sulphonylureas were the most prescribed antihyperglycemic class in 60.4% of patients with HFrEF in our study. The usage of sulphonylurea was frequent despite being associated with hypoglycemia, weight gain, and increased risk of worsening HF (1, 21). Across Asia, the use of sulphonylurea therapies among HFrEF patients ranged from 43.8 to 53% (17, 18). The high prescription rate of sulphonylurea, despite lack of benefits on outcomes, might be due to other factors not examined in our study, such as the presence of renal impairment or hepatic disease, where metformin use is contraindicated. Although the ADVANCE trial had reported no difference in HF hospitalization rate in patients randomized to no-sulphonylurea group or gliclazide-combination group, other studies have found unfavorable risk profile of sulphonylurea when compared with metformin, with sulphonylurea being associated with higher risk of all-cause mortality and congestive HF (22, 23). Hence, a shift from sulphonylurea to metformin prescription should be considered over time. However, if adequate glycemic control cannot be achieved with metformin or other classes of antihyperglycemic agent with regards to contraindications, co-morbidities, or relative cost, sulphonylurea could be used with caution and close monitoring. In addition, patients receiving gliclazide should be given gliclazide modified release (MR) formulation as its once-daily dosing regimen helps to enhance patient's adherence to medication, thus resulting in better blood glucose control when compared to conventional gliclazide.

There was a strikingly under-usage of SGLT-2 inhibitors in our study cohort with a prescription rate of 2.1%, in contrast to 26.5% in Taiwan as reported by Chang HY et al. (18). Although Chang HY et al. reported a higher prescription rate, SGLT-2 inhibitor was only the fourth most commonly prescribed antihyperglycemic agent after metformin, Dpp-4 inhibitor, and sulphonylurea in their cohort of heart failure patients (18). Reason for the infrequent use of SGLT-2 inhibitors in our study cohort could be related to higher cost and limited drug formulary availability. The cost of SGLT-2 inhibitors had also severely limits its availability in other developing countries within Asia (20). The DAPA-HF trial, EMPA-REG OUTCOME trial (Empagliflozin Cardiovascular Outcome Event Trial in Type 2 Diabetes Mellitus Patients), and CANVAS (Canagliflozin Cardiovascular Assessment Study) confirmed that SGLT2- inhibitor treatment lowered rates of heart failure hospitalization (24–26). Because of the consistent cardiovascular benefits demonstrated among both the Asians and non-Asians, it is important to consider escalating the use of SGLT-2 inhibitors in HF patients with T2DM.

In this study, glycemic control was not found to be associated with LVEF. This stands in contrast to the results from a cohort study in a region of South China which observed a curved, U-shaped correlation between HbA1c levels with LV diastolic dysfunction (2, 27). Our study has shown that male was independently associated with lower LVEF. This sex-related differences was consistent with findings from the Registry to Improve the Use of Evidence-Based Heart Failure Therapies in the Outpatient Setting (IMPROVE HF) which revealed that females were significantly associated with improvement in LVEF when compared to males (28). The result from our study indicated that LVEF might be affected by other confounding factors not examined in our study such as BMI, smoking status, co-morbidities or heart failure medications and their doses.

### Study Strength and Limitation

In this study, real-time data such as antihyperglycemic agents, glycemic control, patients' HF classification according to both LVEF and NYHA functional class were collected and analyzed, reflecting clinical practice in the actual situation. The exposure duration of patients to antihyperglycemic agents was well-defined, which was at least 3 months. Research site was selected based on patient population served, HF patient volume, and availability of expertise in echocardiography. There were several limitations in this study. Sampling of subjects from only one hospital implicates that the demonstrated characteristics of this study population might not accurately represent the entire Malaysian population. Also, due to the retrospective nature of the study design, there were incomplete information and missing data from some of the patients' medical records, such as the duration of HF and T2DM, as well as patients' renal and hepatic functions.

## Conclusion

The prescribing patterns of antihyperglycemic agents varied amongst the three LVEF subgroups. Metformin was the most commonly prescribed antihyperglycemic agent. SGLT-2 inhibitor was under-prescribed despite the Malaysian CPG recommendations and evidence that SGLT-2 inhibitor was related to improved outcomes in patients with established HF and DM. Our study also detected poorly-control diabetes presented in the vast majority of patients with heart failure. Understanding the current prescribing pattern of antihyperglycemic agents would help raise awareness of the importance of evidence-based treatment strategies in HF patients with T2DM to improve patients' outcomes. As both HF and DM are chronic disorders, HF patients with T2DM should be managed with close monitoring on safety and efficacy of antihyperglycemic therapies.

Data are scarce regarding the co-disease management strategies and clinical outcomes in HF patients with DM in Malaysia. A prospective follow-up study on LVEF changes and cardiovascular outcomes of hospitalization and mortality in this study population is ongoing to address these gaps. It is hope that the result from the follow-up study will provide further insight into the long-term cardioprotective effects of glucose-lowering medications, thereby enhancing diabetes and heart failure pharmacological management, and optimizing both quality and quantity of life in HF patients with T2DM.

## Data Availability Statement

The raw data supporting the conclusions of this article will be made available by the authors, without undue reservation.

## Ethics Statement

The studies involving human participants were reviewed and approved by The Medical Research and Ethics Committee (MREC), Ministry of Health Malaysia. Written informed consent for participation was not required for this study in accordance with the national legislation and the institutional requirements.

## Author Contributions

SL, HZ, and KC conceived the presented idea. SL developed the methodology and performed the computations. SL and KP collected the data. HZ and KC vetted the study design, data and statistics generated, references, and verified the analytical methods. ST aided in interpreting the results and worked on the manuscript. All authors discussed the results and contributed to the final manuscript.

## Conflict of Interest

The authors declare that the research was conducted in the absence of any commercial or financial relationships that could be construed as a potential conflict of interest.
